# Management of Juvenile Hallux Valgus Deformity: the role of combined Hemiepiphysiodesis

**DOI:** 10.1186/s12891-019-2867-7

**Published:** 2019-10-25

**Authors:** Ming-Hung Chiang, Ting-Ming Wang, Ken N. Kuo, Shier-Chieg Huang, Kuan-Wen Wu

**Affiliations:** 1 Department of Orthopaedic Surgery, National Taiwan University Hospital Hsin-Chu Branch, No.25, Lane 442, Sec.1, Jingguo Rd., Hsinchu City, 300 Taiwan; 2 Department of Orthopaedic Surgery, National Taiwan University Hospital, No.7, Chung Shan S. Rd., Zhongzheng Dist., Taipei City, 10002 Taiwan; 3 Department of Orthopaedic Surgery, School of Medicine, National Taiwan University, No1, Sec. 1, Jen-Ai Rd., Taipei City, 10051 Taiwan; 4 Cochrane Taiwan, Taipei Medical University, No. 250 Wu-Xing Street, Taipei City, 11031 Taiwan; 5 Institute of Biomedical Engineering, National Taiwan University, No1, Sec. 1, Jen - Ai Rd., Taipei, 100 Taiwan

**Keywords:** Juvenile hallux valgus, Hemiepiphysiodesis, Hallux valgus angle, Intermetatarsal angle

## Abstract

**Background:**

This study aimed to investigate the efficacy of percutaneous hemiepiphysiodesis for gradual correction of symptomatic juvenile hallux valgus (HV) deformity.

**Methods:**

Between 2012 to 2014, 24 patients with symptomatic juvenile HV were treated by combined percutaneous medial drilling hemiepiphysiodesis of the first proximal phalanx and lateral transphyseal screw hemiepiphysiodesis of the first metatarsal at our institution. Twenty-one of 24 patients fulfilled inclusion criteria had a complete radiological and clinical follow-up of at least 2 years. Preoperative and postoperative radiographs of the feet were reviewed for measurements of hallux valgus angle (HVA), intermetatarsal angle (IMA), proximal metatarsal articular angle (PMAA), proximal phalangeal articular angle (PPAA), and metatarsal length ratio (MTLR). Clinical outcomes were assessed using the AOFAS hallux metatarsophalangeal-interphalangeal score.

**Results:**

The study included 21 consecutive patients (37 ft) for analysis. The mean age at surgery was 12.0 years (SD = 1.3) and mean follow-up after surgery was 35.1 months (SD = 6.0). With the data available, the HV deformity improved in terms of the reduction of HVA by a mean of 4.7 degrees (*P* < .001) and the reduction of IMA by 2.2 degrees (*P* < .001). The PMAA and PPAA also improved significantly in the anteroposterior plane; however, the PMAA difference was insignificant in lateral plane as expected. The mean difference in the MTLR was 0.00 (*P* = .216) which was indicative of no length discrepancy between first and second metatarsals. The AOFAS score increased from 68.7 to 85.2 (*P* < .001). In correlation analysis, time to physeal closure was significantly correlated with the final HVA change (r = −.611, *P* = .003).

**Conclusion:**

Although combined hemiepiphysiodesis does not create a large degree of correction as osteotomy, yet it did improve HV deformity with adequate growth remaining in our series. It is a procedure that can be of benefit to patients with symptomatic juvenile HV from this minimal operative approach before skeletal maturity.

**Level of evidence:**

Level IV, retrospective case series.

## Background

Juvenile or adolescent hallux valgus (HV), also known as bunions, is a forefoot deformity commonly seen in the skeletally immature population [1, 2]. The deformity initially consists of lateral deviation of the great toe with the apex at the first metatarsophalangeal (MTP) joint, but as the condition progresses it involves the entire forefoot. The etiology and natural course of this disorder have not yet been clearly understood, Coughlin et al. reported a strong maternal family history [3]. Symptoms may include painful erythematous bunion, unsatisfactory cosmesis, and difficult footwear fitting.

The management of HV deformities in skeletally immature patients remain controversial, either conservative or operative treatments. With uncertainty of halting progression in conservative management, operative intervention is often required in symptomatic skeletally immature HV [2, 4]. However, traditional operative options, such as proximal or distal first metatarsal osteotomy, the recurrence rates have been reported at a range of 20–40% in young patients with open physis, leading to an undesirable outcome [5–7]. Accordingly, some authors advocated to postpone surgery through first metatarsal osteotomy with or without soft tissue balancing procedures until skeletal maturity [8].

The hemiepiphysiodesis in skeletally immature patients, by tethering marginal physis and creating asymmetrical physeal growth, has been used for treatment of angular deformities of the lower limbs for many years [9]. The potential advantages of hemiepiphysiodesis are less invasive, minimal scarring, and short hospital stay. Lateral hemiepiphysiodesis of the first proximal metatarsus alone has been proposed by a few previous series to correct the metatarsus varus component in juvenile HV deformity [10, 11]. However, the smaller sample size in previous series was difficult to demonstrate the sequential angular changes and identify factors influencing the efficiency of the hemiepiphysiodesis technique after surgery.

We hypothesize that combined hemiepiphysiodesis targeting the first ray proximal phalangeal and metatarsal physis is effective in management of juvenile HV with adequate growth remaining. The aims are to investigate the efficacy and the complication associated with the combined procedures and the factors related to the operative outcomes.

## Methods

We retrospectively reviewed the case series of all patients with juvenile HV (HV angle >16 degrees) who received combined hemiepiphysiodesis surgery from 2012 to 2014 at our institution. Pre-operatively, all included patients complained of pain, redness, or callosity at the bunion with failed conservative treatment, including analgesics, shoe modification or orthotics. Other requirements for inclusion were patients with a minimum of 2 years of complete radiographic and clinical follow-up. Patients with neuromuscular disease, juvenile rheumatoid arthritis and connective tissue disorders were eliminated. The medical records were reviewed for chief complaints, age at surgery, length of follow-up, the need for future foot surgeries, and complications. The study has been approved by the Research Ethics Committee of National Taiwan University Hospital (201601015RIND).

### Radiographic measurements

**Fig. 1:**
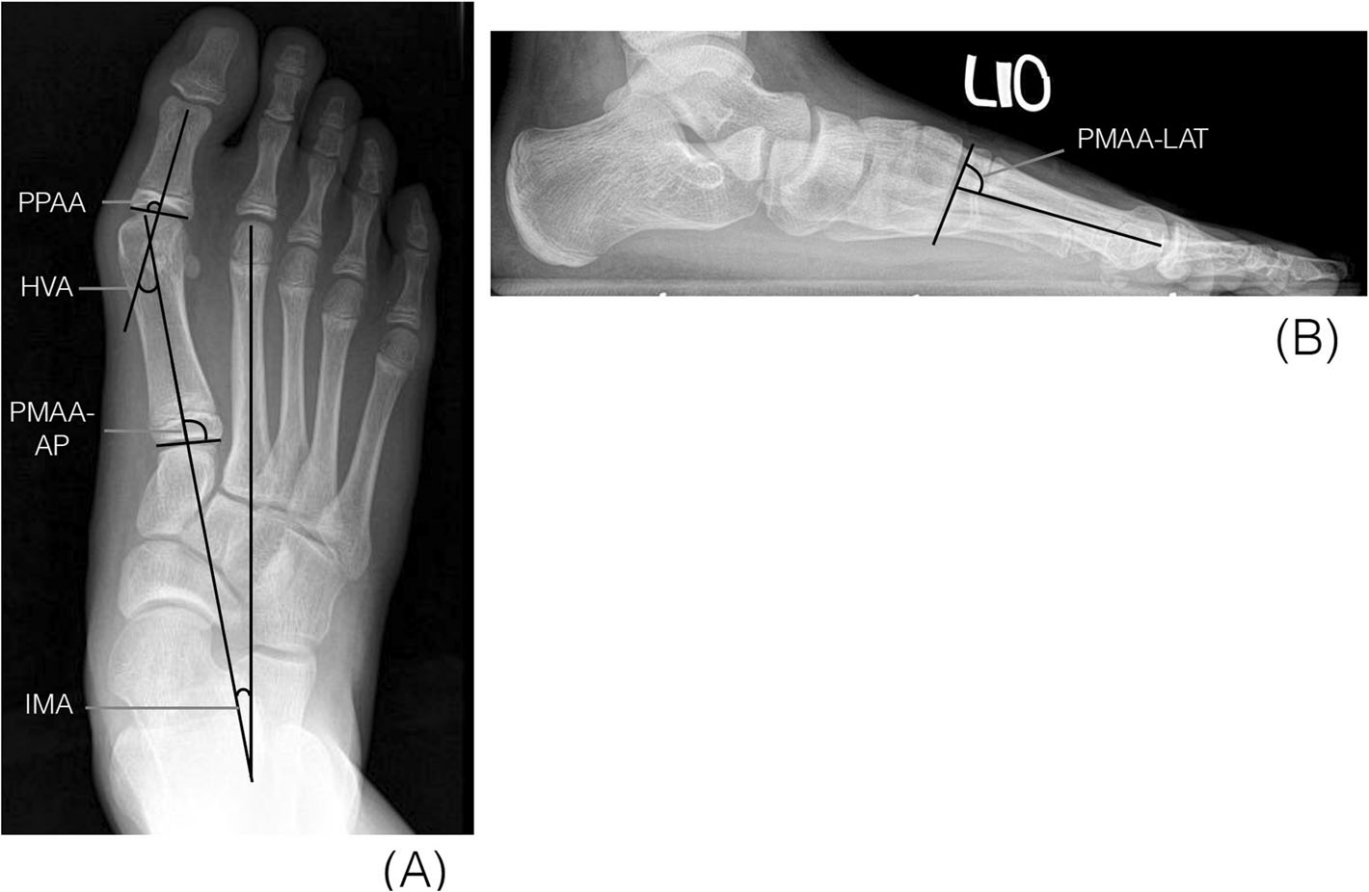
Radiographic angle measurements: (**a**) Intermetatarsal angle (IMA), hallux valgus angle (HVA), proximal metatarsal articular angle-AP (PMAA-AP), proximal phalangeal articular angle (PPAA) (**b**) proximal metatarsal articular angle (PMAA-LAT)

**Fig. 2:**
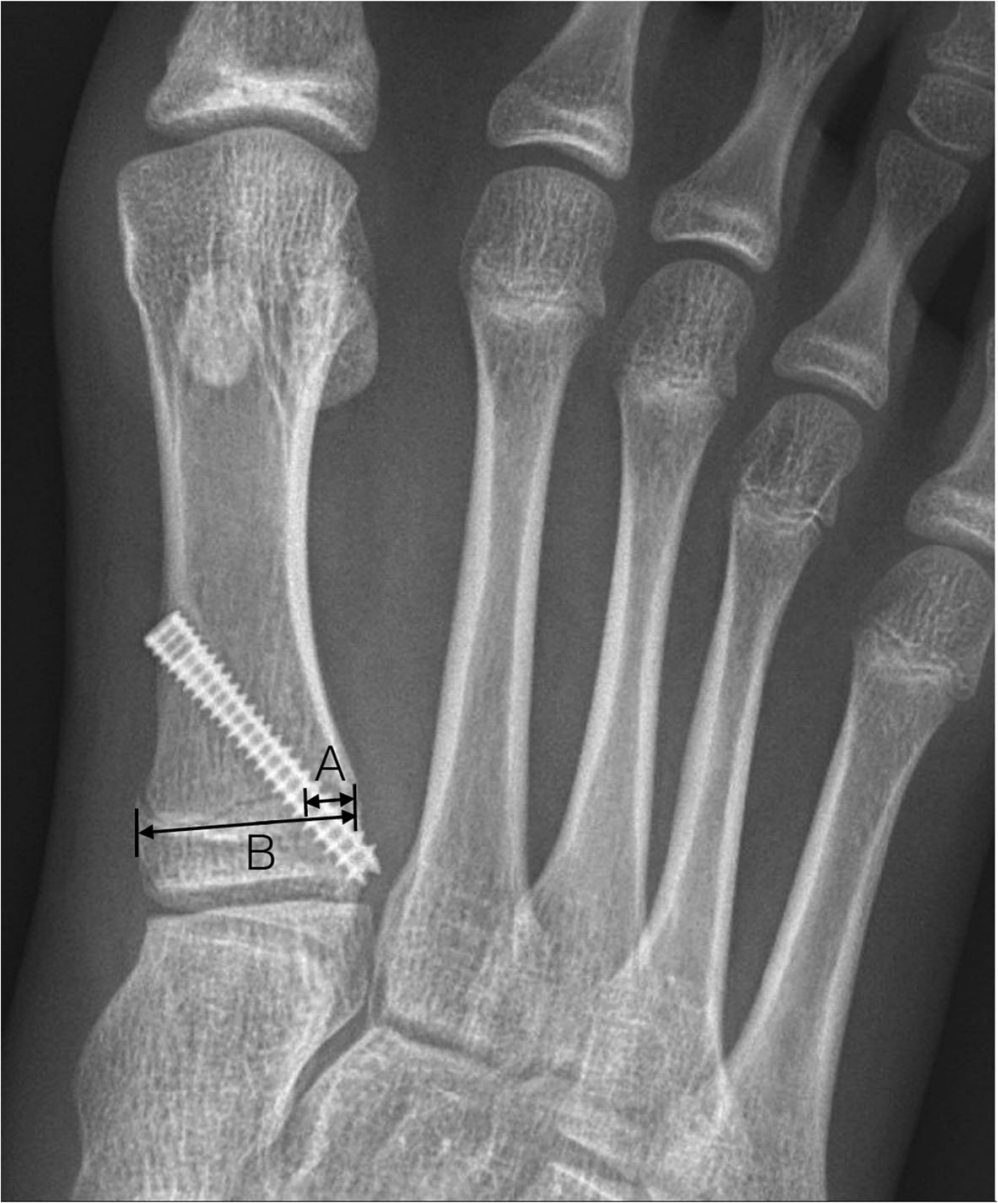
Line A: from center of screw at physeal crossing to lateral border of physis parallel to line B. Line B: length of proximal physis, “A/B” represents screw position

All radiographs of the feet were taken in standard weight-bearing anterior-posterior (AP) and lateral view of foot before surgery and at 3 to 6-month interval postoperatively until the latest follow-up. All patients had their physis closed at final follow up and the time from surgery to radiographic physeal closure was recorded. We measured the hallux valgus angle (HVA), intermetatarsal angle (IMA), proximal metatarsal articular angle (PMAA), proximal phalangeal articular angle (PPAA) (Fig. 1a & b), the metatarsal length ratio (MTLR) and the screw position relative to physis [10, 12, 13]. The PMAA and PPAA were determined by the intersection of the bone’s long axis and the line along its proximal articular surface of the first metatarsal and proximal phalanx for investigation of any alignment changes after hemiepiphysiodesis. The distal-lateral angle and the distal-dorsal angle of the first metatarsal were defined as PMAA-AP and PMAA-LAT, respectively. The distal-medial angle of the first proximal phalanx was defined as PPAA. The MTLR was developed to assess the relative length ratio of the first and second metatarsals to assess possible shortening of the first metatarsal after lateral hemiepiphysiodesis. In order to analyze if the screw position was an influential factor, we measured the length of first metatarsal physis (B) and the distance from center of the screw that crossing the physis to lateral border of the physis (A) in the AP foot weight-bearing view. The ratio of A/B was defined as the screw position-AP (Fig. 2).

For the reliability test of radiographic measuring, Intraclass Correlation Coefficient (ICC) was used to analyze the intra-rater and inter-rater reliabilities. Regarding intra-rater reliability, the measurement of above parameters was repeated at 1-week interval by a junior author using AGFA-Orthopaedic-Tools Version 2.10 (Agfa HealthCare N.V. Septestraat 27, B-2640 Mortsel, Belgium). For inter-rater reliability, two authors measured the above parameters independently using the same software.

### Functional assessments

The functional assessments were evaluated with the American Orthopaedic Foot & Ankle Society (AOFAS) hallux metatarsophalangeal-interphalangeal score which comprised of nine questions and cover three categories: Pain (40 points), function (50 points) and alignment (10 points). These are all scored together for a total of 0 to 100 points [14]. All functional and clinical outcomes were assessed in all patients preoperatively and at the final follow-up visit.

### Operative technique and postoperative care

**Fig. 3:**
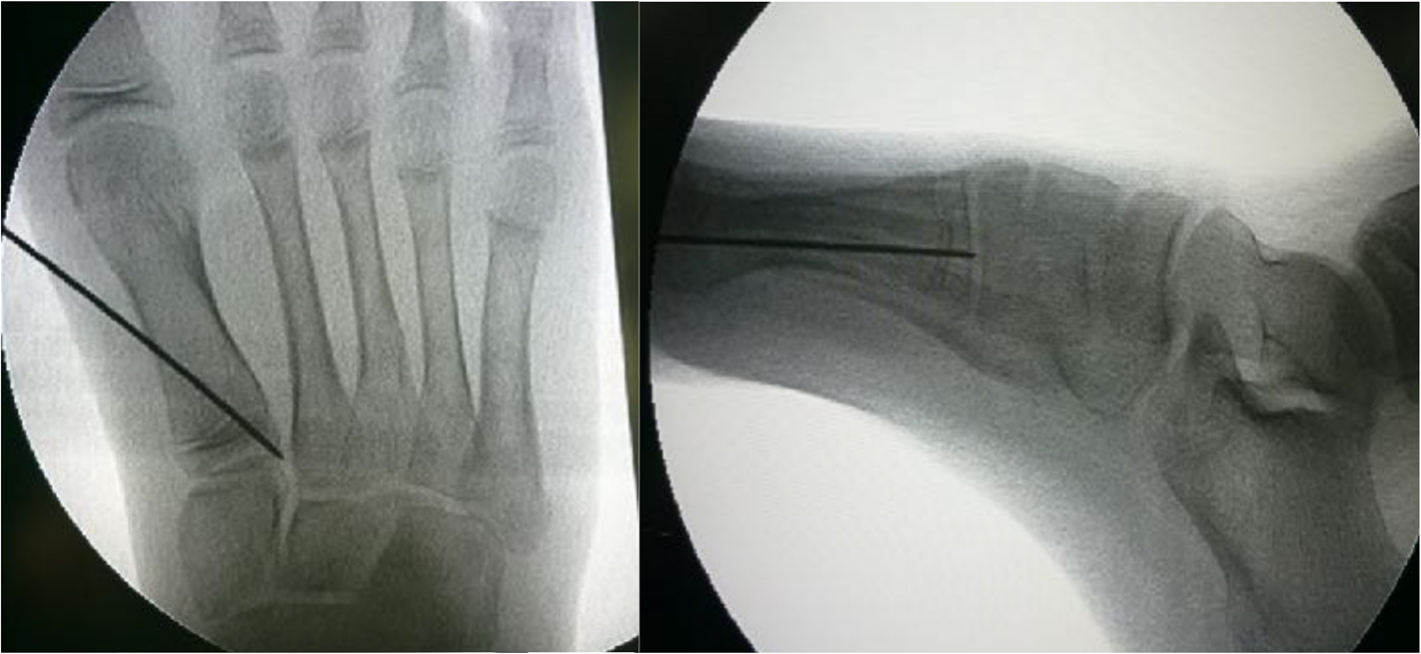
Under fluoroscopic guidance, a guide wire is inserted from the medial cortex distal to the first metatarsal physis, directing to lateral third of the physis in AP view and center of the physis in lateral view

All procedures were carried out identically by the two senior authors who worked as a team. With patient on supine position under general anesthesia and fluoroscopic control, a guided wire for the cannulated screw is positioned retrograde from the medial cortex of first metatarsal mid-shaft and pointed toward the lateral and proximal corner of first metatarsus. The guided wire is ideally crossed through the lateral quarter of first metatarsal physis in AP view and centered of physis in lateral view (Fig. 3). After gauging the length, a cannulated drill is used to make a tunnel and then one 4.0-mm transphyseal screw is inserted (Acutrak, Acumed®, Oregon, USA). For medial hemiepiphysiodesis of first proximal phalanx, a guide wire is inserted through the medial quarter of first proximal phalangeal physis in dorsal-plantar direction. A stab wound is made over the pin entry and a cannulated drill is used to drill over the physis. A curved curette is then inserted into the drilled path for curettage of the surrounding physeal bone. Patients are allowed full weight-bearing ambulation immediately.

### Statistical analysis

All of the parameters were checked for normality first using the Shapiro-Wilk test. Descriptive statistics were given as mean ± standard deviation (SD). The paired sample t-test was used for comparison of the preoperative and the latest radiographic results following index surgery. Pearson’s correlation test was used to analyze the relationship between the final change of HVA and preoperative demographic variables including age at surgery, time to physeal closure and radiographic parameters. All statistical analyses were performed using SPSS software, version 17.0 (SPSS, Inc., Chicago, IL, USA), and a *p* value of < 0.05 was defined to be statistically significant.

## Results

During the period, 43 ft of 24 patients had index procedures for symptomatic juvenile HV. Four feet from 2 patients with cerebral palsy were excluded. There was one patient who did not return for 2 years follow-up. A total of 37 ft in 21 patients (9 boys and 12 girls) met our inclusion criteria for subsequent analysis. The mean age at surgery was 12.0 (SD = 1.3) years. The mean age of the girls and boys at time of surgery were 11.2 (SD = 0.9) years and 13.0 (SD = 1.1) years respectively. Mean follow-up after surgery was 35.1 (SD = 6.0) months. The mean time period from surgery to physeal closure radiographically was 15 (SD = 4.8) months. The average screw position-AP was 0.25 (SD = 0.07) which indicated the position of all screws crossing was consistently located at lateral quarter of the first metatarsal physis in the coronal plane. The screw position-LAT was 0.56 (SD = .07). The demographic data were summarized in Table 1.

**Table 1:** Demographic data

	Results	SD	Range
No. of feet	37	N/A	N/A
Gender (M/F)	9/12	N/A	N/A
Laterality (right/left)	17/20	N/A	N/A
Mean age at surgery (years)	12.0	1.3	9.5~14.5
Mean length of follow-up (months)	35.1	6.0	26.9~51.1
Mean time to physeal closure (months)	15	4.8	8.9~26.2
Screw position-AP	0.25	0.07	0.11~0.40
Screw position-LAT	0.56	0.07	0.41~0.67

### Radiographic outcomes

The changes in the radiographic measurements of the 37 ft after great toe proximal phalangeal and metatarsal hemiepiphysiodesis were summarized in Table 2. The mean HV angle preoperatively for all feet was 25.1 degrees and reduced to 20.4 degrees at the final follow-up. The mean correction in the HVA was 4.7 degrees with statistical significance (*P* < .001). The HVA improved in 33 of 37 ft. Four feet in 4 patients had slight progression of HVA at final follow-up. The IMA of treated feet also significantly improved by a mean of 2.2 degrees (*P* < .001). These feet had a mean preoperative IMA of 12.3 degrees and reduced to a mean final IMA of 10.0 degrees. The IMA improved in 32 of 37 ft. The mean correction of PMAA-AP and PPAA were 2.5 degrees (*P* = .004) and 1.9 degrees (*P* < .001), respectively. These feet had a mean preoperative PMAA-AP of 91.9 degrees and decreased to final of 89.4 degrees. The mean change of the metatarsal sagittal alignment (PMAA-LAT) was 0.2 degrees upward without statistical significance (*P* = .564). The mean change in the MTLR was too small without statistical significance (*P* = .216). The ICC for intra- and inter-rater reliability in all radiographic measurements was greater than 0.80. (Table 2).

**Table 2:** Summary of Radiographic Measurements and Functional Assessments

	Pre-OP		Post-OP		Difference				Intra-rater reliability^c^	Inter-rater reliability^c^
	Mean	SD	Mean	SD	Mean	95% CI	SD	*P* value		
HVA	25.1	4.8	20.4	6.3	−4.7	−6.1~ − 3.3	4.1	<0.001*	0.969	0.916
IMA	12.3	2.4	10.0	2.7	−2.2	−2.9~ − 1.6	2.0	<0.001*	0.914	0.876
PMAA-AP	91.9	5.0	89.4	6.6	−2.5	−4.1~ − 0.9	4.9	0.004*	0.941	0.917
PPAA	97.4	2.7	95.4	2.8	−1.9	−2.7~ − 1.2	2.2	<0.001*	0.897	0.813
PMAA-LAT	86.0	1.9	85.8	1.8	−0.2	−0.7~0.4	1.6	0.564	0.816	0.859
MTLR	0.83	0.03	0.83	0.04	0.00	−0.01~0.01	0.02	0.216	0.922	0.940
AOFAS	68.7	10.1	85.2	12.3	16.5	13.9~19.1	7.8	<0.001	–	–

*HVA* hallux valgus angle, *IMA* intermetatarsal angle, *PMAA-AP* proximal metatarsal articular angle in AP view, *PPAA* proximal phalangeal articular angle, *PMAA-LAT* proximal metatarsal articular angle in lateral view, *MTLR* 1st/2nd metatarsal length ratio, *CI* confidence interval. * *P* < 0.05 in Paired Samples T-test. ^c^ Correlation Coefficient. SD: Standard deviation

Several preoperative clinical factors were analyzed with correlation test to identify potential relationships with the amount of final HVA change. The results are summarized in Table 3. Patients with longer time to physeal closure was associated with larger difference of final HVA correction (r = −.611, *P* = .003).

**Table 3:** Pearson Correlation Coefficients (r) for the Relation of Final HVA Change and Pre-operative Parameters

Variables	r	*P* value
Age	−0.133	0.556
Time to physeal closure	−0.611	0.003^*^
Pre-OP HVA	−0.005	0.977
Pre-OP IMA	0.214	0.204
Pre-OP PPAA	−0.312	0.060
Pre-OP PMAA-AP	0.271	0.104
Pre-OP MTLR	0.266	0.112

*P < 0.05

### Functional outcomes

At the final visit, the AOFAS hallux metatarsophalangeal-interphalangeal scores significantly increased from 68.7 to 85.2 (*P* < .001). All patients had rapid return to school and sports activities. There was no known complication or overcorrection. No patients required revision surgery for residual HV after index procedure.

## Discussion

**Fig. 4:**
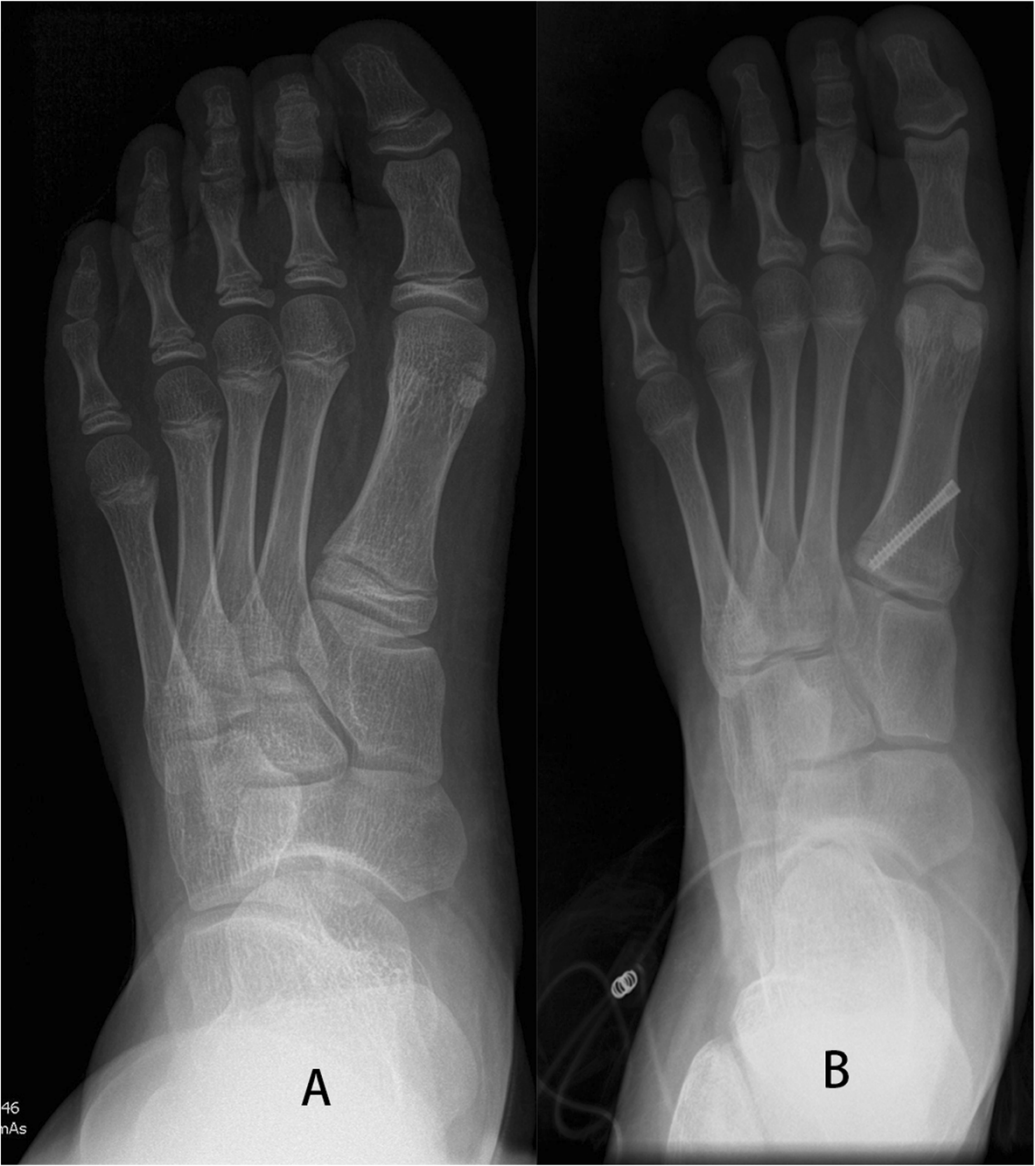
**a** The case example shows the initial HVA was 25.5 degrees, and IMA was 8.5 degrees at age of twelve. **b** Two years and 2 months after the operation, the HVA improved to 14.6 degrees, and IMA improved to 4.7 degrees

Our results showed significant correction of HV deformity (Fig. 4). Clinical symptoms can be relieved in these pediatric population following index procedures. In 2007, Davids et al. reported significant correction of both the HVA and IMA. It was achieved in 55% of 11 ft without worsening of either angle. They concluded that lateral hemiepiphysiodesis of the first metatarsal is a reasonable alternative for management of symptomatic or progressive juvenile HV [10]. The current study showed comparable results, and higher percentage of patients had significant HVA correction (89%, 33 of 37).

Treatment for juvenile HV has always been a challenging problem because of its unclear etiology, pathophysiology, and growing process. Operative correction of symptomatic HV deformities is often recommended once conservative treatment has failed. Unlike the adult population, the high recurrence rate is still a major concern in the treatment of skeletally immature HV by conventional metatarsal osteotomy techniques [5, 6, 15]. An open metatarsal physis has been believed as a predisposing factor to the recurrence risk. Despite innovated technique and methods of fixation have improved the operative outcomes and lowered recurrence rate to 8% based on a recent systematic review article, most of the series included were focused on the adolescent rather than juvenile population [2, 15]. Temporary maintenance with orthotics and postponing surgery until mid- to late teens is usually preferred for skeletally immature HV. However, study of the natural history showed juvenile HVA might increase by 0.8 degrees per year [16]. Our study revealed a reversed trend of the HVA progression. (Table 4).

**Table 4:** Comparison of Current Study and Natural Course Study (Sung et al)

	Current Study	Sung et al
Age at first visit (years)	12.0	10.2
HVA at first visit	25.1	18.4
IMA at first visit	12.3	10.1
Follow-up duration (years)	2.9	2.8
Progression rate of HVA (degrees/year)	−1.8	0.8
Progression rate of IMA (degrees/year)	−0.8	0.0

*HVA* hallux valgus angle, *IMA* intermetatarsal angle

Negative value in progression rate indicative of improvement

Most of conventional operative modalities neglect the potential of manipulating the physeal growth of the first metatarsal and proximal phalanx to stop deformity progression and allow gradual correction of skeletal deformity. A small number of studies have suggested the lateral hemiepiphysiodesis of the first metatarsal is a reasonable alternative for the symptomatic or progressive juvenile HV [10, 11]. However, prior techniques used for lateral hemiepiphysiodesis, either curettage or stapling, could not compare with percutaneous manner in simplicity and effectiveness.

A temporary epiphysiodesis using transphyseal screw, described by Metaizeau et al. [9] and then by Khoury et al. [17], is a true percutaneous technique and effective for limb length discrepancy and angular deformities correction. In our strategy, a simple stab wound each can achieve hemiepiphysiodesis in both locations, provides immediate ambulation. The transphyseal screw technique also carries a theoretical advantage of reversibility compares to traditional physeal ablation technique [17].

Although the potential for correction is not as powerful as osteotomies based on prior studies, ranging from 7.3 degrees to 21.54 degrees change in HVA, and 0.87 to 9.25 degrees change in IMA [7, 15, 18–24], osteotomy procedures also carry higher complication rates (22.9%), such as metatarsal shortening, first metatarsal-phalangeal (MTP) joint stiffness, and recurrent HV deformity [2]. Shortening or dorsiflexion of the first ray are common complications of HV osteotomy and may result in transfer metatarsalgia [25]. In the other way, our result didn’t show significant change in metatarsal length ratio (MTLR) or sagittal alignment (PMAA-LAT).

Despite of hemiepiphysiodesis, the deformity may still progress for reasons including timing of surgery, quality of surgery, and underlying cause of deformity. Four feet in 4 patients in this study had slight progression of HVA. One foot might have the screw placed too laterally (a screw position AP of 0.12); therefore, less threads can purchase epiphysis. The failure of other 3 ft might be attributed to late timing for surgery (less than 9 months before permanent physeal closure). From our analysis, time to physeal closure was correlated with the final HVA change. Although there is still no consensus on the optimal timing of metatarsal hemiepiphysiodesis, previous studies suggested that adequate growth remaining to achieve significant correction by hemiepiphysiodesis is up to 10 years for girls and up to 12 years for boys [26]. The mean age at surgery in our series was 11.2 years for girls and 13.0 years for boys which were relative older than prior studies. According to the growth chart by Nelson [26], the estimated growth of the first metatarsal is only 8~10% remaining in our study group. At this late stage of foot growth, it is unlikely to cause significant shortening of the first metatarsal by our procedures, but the power of angular correction may not be as adequate. Consequently, proximal phalangeal hemiepiphysiodesis was added as a supplementary procedure to augment HV correction. For optimum results, this surgery may be performed a few months earlier than the operative age in current series. The authors agreed with Davids et al. [10] that the best timing of the index procedure should be performed with 2 or more years of growth remaining.

This study had two limitations. First, this is a retrospective case series study with limited sample size. Second, this study didn’t directly compare with untreated patients with juvenile HV. In spite of this, most of the patients in this study had improvement in HVA and IMA, which were better than those following their natural history [16].

## Conclusions

With adequate growth remaining, the combined metatarsal and proximal phalangeal hemiepiphysiodesis is a simple and effective procedure for juvenile HV patients with minimal approach. Although combined hemiepiphysiodesis does not create a large degree of correction as osteotomy, the percutaneous procedures greatly reduce the postoperative disability with immediate weight-bearing. The metatarsal length ratio and the sagittal alignment of the first metatarsal were retained. Longer follow-up is needed to observe possible recurrence of the deformity. In the future, a head-to-head comparison with non-surgical cases would help to draw firm conclusions.

## Data Availability

The datasets used and/or analyzed during the current study are available from the corresponding author on reasonable request.

## Abbreviations

HVA: hallux valgus angle
IMA: intermetatarsal angle
MTLR: metatarsal length ratio
PMAA: proximal metatarsal articular angle
PPAA: proximal phalangeal articular angle
SD: standard deviation
